# Skeletal muscle microvascular perfusion responses to cuff occlusion and submaximal exercise assessed by contrast‐enhanced ultrasound: The effect of age

**DOI:** 10.14814/phy2.14580

**Published:** 2020-10-10

**Authors:** Annelise L. Meneses, Michael C. Y. Nam, Tom G. Bailey, Chris Anstey, Jonathan Golledge, Michelle A. Keske, Kim Greaves, Christopher D. Askew

**Affiliations:** ^1^ VasoActive Research Group School of Health and Sport Sciences University of the Sunshine Coast Maroochydore QLD Australia; ^2^ Department of Cardiology Sunshine Coast University Hospital Birtinya QLD Australia; ^3^ Centre for Research on Exercise, Physical Activity and Health School of Human Movement and Nutrition Sciences The University of Queensland Brisbane QLD Australia; ^4^ Department of Intensive Care Sunshine Coast University Hospital Birtinya QLD Australia; ^5^ Queensland Research Centre for Peripheral Vascular Disease College of Medicine and Dentistry James Cook University Townsville QLD Australia; ^6^ Department of Vascular and Endovascular Surgery The Townsville Hospital Townsville QLD Australia; ^7^ School of Exercise and Nutrition Sciences Institute for Physical Activity and Nutrition Deakin University Geelong VIC Australia; ^8^ Sunshine Coast Health Institute Sunshine Coast Hospital and Health Service Birtinya QLD Australia

**Keywords:** aging, blood flow, exercise, muscle perfusion, reactive hyperemia, vascular conductance

## Abstract

Impairments in skeletal muscle microvascular function are frequently reported in patients with various cardiometabolic conditions for which older age is a risk factor. Whether aging per se predisposes the skeletal muscle to microvascular dysfunction is unclear. We used contrast‐enhanced ultrasound (CEU) to compare skeletal muscle microvascular perfusion responses to cuff occlusion and leg exercise between healthy young (*n* = 12, 26 ± 3 years) and older (*n* = 12, 68 ± 7 years) adults. Test–retest reliability of CEU perfusion parameters was also assessed. Microvascular perfusion (microvascular volume × flow velocity) of the medial gastrocnemius muscle was measured before and immediately after: (a) 5‐min of thigh‐cuff occlusion, and (b) 5‐min of submaximal intermittent isometric plantar‐flexion exercise (400 N) using CEU. Whole‐leg blood flow was measured using strain‐gauge plethysmography. Repeated measures were obtained with a 15‐min interval, and averaged responses were used for comparisons between age groups. There were no differences in post‐occlusion whole‐leg blood flow and muscle microvascular perfusion between young and older participants (*p* > .05). Similarly, total whole‐leg blood flow during exercise and post‐exercise peak muscle microvascular perfusion did not differ between groups (*p* > .05). The overall level of agreement between the test–retest measures of calf muscle perfusion was excellent for measurements taken at rest (intraclass correlation coefficient [ICC] 0.85), and in response to cuff occlusion (ICC 0.89) and exercise (ICC 0.95). Our findings suggest that healthy aging does not affect muscle perfusion responses to cuff‐occlusion and submaximal leg exercise. CEU muscle perfusion parameters measured in response to these provocation tests are highly reproducible in both young and older adults.

## Graphical abstract

We used contrast‐enhanced ultrasound (CEU) to compare skeletal muscle microvascular perfusion responses to cuff occlusion and leg exercise between healthy young (*n* = 12, 26 ± 3 years) and older (*n* = 12, 68 ± 7 years) adults. There were no differences in post‐occlusion whole‐leg blood flow and muscle microvascular perfusion between young and older participants (*p* > .05). Similarly, total whole‐leg blood flow during exercise and post‐exercise peak muscle microvascular perfusion did not differ between groups (*p* > .05).

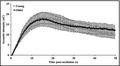

## 1 INTRODUCTION

Skeletal muscle microvasculature plays a critical role in the capillary‐tissue exchange of oxygen and nutrients. There is evidence of a reduction in skeletal muscle capillary blood flow (perfusion) during and following exercise in people with various age‐related chronic conditions, such as type 2 diabetes (Groen et al., 2014; Sacre et al., 2015), peripheral arterial disease (Davidson et al., 2017; Kundi et al., 2017), and chronic heart failure (Copp et al., 2010; McDonough et al., 2004). Whether this microvascular impairment is a result of the disease alone or affected by older age is not fully understood.

Age‐related reductions in the capillary density of the leg muscles have been reported in some (Coggan et al., 1992; Frontera et al., 2000; Groen et al., 2014; Ryan et al., 2006); but not all previous studies (Chilibeck et al., 1997; Hildebrandt et al., 2017). Ultrastructural changes, including an increase in the pericapillary basement membrane thickness (Bigler et al., 2016; Scelsi et al., 1980) and a loss of endothelial cells in skeletal muscle capillaries, have also been reported in elderly compared with young adults (Bigler et al., 2016; Delp et al., 2008). These structural capillary changes may reduce the capacity to adequately support muscle tissue insulin, glucose, and oxygen uptake (Kusters & Barrett, 2016; Poole et al., 2013), and limit exercise capacity. However, the extent to which these age‐related changes impair skeletal muscle microvascular function and perfusion in vivo is unclear.

Contrast‐enhanced ultrasound (CEU) imaging allows for the quantification of microvascular perfusion in skeletal muscle at rest and in response to high‐flow demand provocation, such as during or following exercise (Hildebrandt et al., 2017; Lindner et al., 2008) and in response to post‐occlusion reactive hyperemia (Amarteifio et al., 2012; Thomas et al., 2014). Previous studies that have used CEU to investigate the effect of age have reported inconsistent findings where some (Durham et al., 2010; Thomas et al., 2014), but not all (Hildebrandt et al., 2017) have reported a deficit in microvascular blood flow in older adults compared with young. Each of these studies has used a different method of CEU imaging (e.g., bolus vs. continuous contrast delivery, intermittent vs. real‐time imaging), and different provocation stimuli including a short period of thigh‐cuff occlusion (Thomas et al., 2014), submaximal treadmill walking (Durham et al., 2010), and isometric knee extension exercise (Hildebrandt et al., 2017). It is presently unclear whether the muscle microvascular responses are a consequence of microvascular dysfunction, or whether they reflect differences in other factors, such as whole‐leg blood flow, leg muscle‐mass, or intensity of the stimuli, for example, exercise intensity, which could all possibly cloud the influence of age on skeletal muscle perfusion (Donato et al., 2006; Reilly et al., 2018).

The diverse findings reported in previous studies might also be attributed to a lack of standardization in image acquisition and analysis procedures, and consequently the poor reproducibility of microvascular perfusion assessment using CEU (Lindner et al., 2008; Thomas et al., 2014). Recent CEU studies have adopted a more consistent approach and calculated microvascular blood flow parameters by fitting image intensity data to an exponential response curve (Davidson et al., 2017; Hildebrandt et al., 2017; Sacre et al., 2015); however, the reliability of these parameters remains to be determined. Therefore, this study aimed to compare the skeletal muscle microvascular perfusion and whole‐leg blood flow responses to standardized cuff occlusion and matched submaximal intensity exercise protocols between young and older participants. The secondary aims were to explore the relationship between the microvascular perfusion and whole‐leg blood flow responses and to assess the test–retest within‐day reliability of CEU skeletal muscle perfusion parameters.

## 2 MATERIALS AND METHODS

### 2.1 Participant recruitment

Twelve apparently healthy elderly (68 ± 7 years) and twelve healthy young (26 ± 3 years) participants were recruited through local community announcements and media releases. The healthy older participants also served as a control group in a previously published study of patients with peripheral arterial disease from our group, which was conducted at the same time (Meneses et al., 2018). Prior to participation, participants were screened for cardiovascular and metabolic diseases using a medical history questionnaire and physical examination, including ankle‐brachial index measurement, and a fasting blood sample was taken for hematological and blood lipid analyses. Participants were excluded if they were current smokers, had type 2 diabetes, heart or cerebrovascular diseases, uncontrolled hypertension (systolic blood pressure >180 mm Hg or diastolic blood pressure >110 mm Hg), were taking hormone replacement therapy (post‐menopausal older females) or participated in competitive sport or supervised exercise training. All participants gave written informed consent to participate in the study, which was approved by the Prince Charles Hospital (HREC/14/QPCH/122) and University of the Sunshine Coast (A/15/706) Human Research Ethics Committees. All procedures were conducted in accordance with the Declaration of Helsinki.

### 2.2 Study overview

Participants initially underwent screening and baseline measures and completed a seven‐day physical activity recall (Australian Bureau of Statistics, 2011/2012). Participants were familiarized with the plantar‐flexion exercise protocol and performed a maximum force test. Participants attended an experimental session (session 1) for the assessment of whole‐leg blood flow at rest and following thigh‐cuff occlusion using strain‐gauge plethysmography. Then, contraction‐by‐contraction whole‐leg blood flow was recorded during a 5‐min bout of intermittent isometric plantar‐flexion exercise. Participants attended two further experimental sessions, on separate days, where muscle microvascular perfusion of the medial gastrocnemius was assessed using CEU before and after 5‐min of thigh‐cuff occlusion (session 2), and before and after the same 5‐min plantar‐flexion exercise protocol (session 3), as previously described (Meneses et al., 2018). Within each session, test procedures (cuff occlusion or exercise) were conducted twice, separated by 15 min, for the assessment of within‐day reliability, and average responses were used for the comparison of microvascular responses between age groups. All measurements were performed in the non‐dominant limb of the participants. Experimental sessions were held at the same time of day, at least 72 hr apart, and participants were instructed to avoid exercise and caffeine or alcohol consumption for 24 hr before each session. Young females were evaluated during the midfollicular phase of their menstrual cycle (days 5–12).

### 2.3 Resting blood pressure and ankle‐brachial index

Resting blood pressure and ankle‐brachial index (ABI) were measured in triplicate to screen for uncontrolled hypertension and peripheral arterial disease. Resting brachial blood pressure was measured using an automated blood pressure monitor (GE Medical Systems Information Technologies, Inc) after participants were supine for at least 10 min. Resting ankle systolic blood pressure was measured using a handheld Doppler ultrasound probe (Bi‐directional Doppler MD6, Hokanson Inc.) and sphygmomanometer. To confirm the absence of peripheral arterial disease, the ABI of each leg was calculated as the higher ankle pressure (dorsalis pedis or posterior tibial artery) divided by the higher brachial pressure (left or right arm) as previously described (Askew et al., 2002).

### 2.4 Anthropometric assessments

Height, weight, and body mass index (BMI) were determined using standardized anthropometric measures. Estimated calf muscle mass (ECMM) was determined as: ECMM = (TL × 38.20851 + (LG − 3.14259 × (SF/10)) × 80.24425 − 2,467.9)/1,000; where ECMM = estimated calf muscle mass (kg), TL = tibial length (cm), LG = calf girth (cm) and SF = average of the closest two out of three skinfolds of the mid‐calf (mm), as previously described (Clarys & Marfell‐Jones, 1986). Calf girth and tibial length were recorded and used as landmarks for the standard positioning of the strain‐gauge and ultrasound probe during experimental sessions.

### 2.5 Plantar‐flexion exercise

Participants were seated upright with their hip and knee flexed (~90°) and their foot on an immovable footplate. A calibrated load cell (Xtran S1W, Applied Measurement) was positioned over the distal‐thigh, and in this position, participants performed unilateral intermittent isometric plantar‐flexion contractions. Contraction force was displayed on a monitor enabling participants to regulate their effort. After being familiarized with the exercise, participants performed five maximum voluntary contractions (MVC), each separated by 60 s rest, and the average was used as the measure of maximum force. The 5‐min bouts of intermittent isometric plantar‐flexion exercise (sessions 2 and 3) were performed at a fixed contraction intensity of 400 N and at a fixed contraction frequency (5‐s duty cycle: 2‐s contractions separated by 3‐s relaxation).

### 2.6 Whole‐leg blood flow

Whole‐leg blood flow was measured using strain‐gauge plethysmography (EC6 Plethysmograph, Hokanson, Inc.). A rapid inflation blood pressure cuff (CC17 contored leg cuff, E20 Rapid Cuff Inflator, and AG101 Cuff Inflator Air Source, Hokanson Inc.) was placed around the upper thigh, and a mercury‐in‐silastic strain‐gauge was placed around the calf at the largest circumference for the assessment of limb volume changes. Resting measures were obtained by inflating the cuff to 60 mm Hg for 10 s, with blood flow assessed as the rate of rise in limb volume over two cardiac cycles following cuff inflation. Resting measurements were performed in triplicate, separated by 20 s, and the average was used for analysis. Post‐occlusion whole‐leg blood flow was measured by inflating the thigh cuff to 200 mm Hg for five minutes to completely occlude arterial blood flow. The cuff was then rapidly deflated to the venous occlusion pressure (60 mm Hg), and blood flow was measured over two cardiac cycles following cuff deflation (Hokanson et al., 1975).

#### 2.6.1 Exercise whole‐leg blood flow and vascular conductance

Contraction‐by‐contraction leg blood flow and vascular conductance were measured throughout exercise using strain‐gauge plethysmography, using a similar approach to that described previously (Egana & Green, 2005; Murphy et al., 2018). This technique has been shown to provide a valid estimate of the hyperemic response during intermittent plantar‐flexion exercise compared with Doppler ultrasound (Green et al., 2011). Five minutes before the commencement of exercise, pre‐exercise seated whole‐limb blood flow was measured in triplicate and averaged. A thigh cuff was inflated to 30 mm Hg for the duration of the exercise. This pressure was chosen to occlude venous return without interfering with arterial blood flow (Askew & Matthews, 2012). Blood flow was assessed following each contraction as the change in limb volume over the first complete cardiac cycle, free of any movement artifact, from the onset of the 3‐s relaxation phase. Single‐lead electrocardiogram, heart rate (ADInstruments), and beat‐by‐beat finger blood pressure (Finometer, Finapres Medical Systems) were continuously monitored during exercise. Leg vascular conductance was calculated at rest and following each contraction as leg blood flow divided by mean arterial pressure.

Contraction‐by‐contraction leg blood flow and vascular conductance were averaged across the repeated within‐session trials and fitted to a biphasic exponential response curve (Table Curve 2D v4, SPSS Inc.) to determine the total rise in whole‐leg blood flow during exercise, as previously described (Reeder & Green, 2012; Saunders et al., 2005). The goodness of fit for the whole‐leg blood flow and vascular conductance data, based on the adjusted *R* ^2^, was 0.83 ± 0.10 and 0.79 ± 0.10, respectively.

### 2.7 Calf muscle microvascular perfusion

Calf muscle microvascular perfusion was measured using CEU. During experimental session 2, CEU imaging of the medial gastrocnemius was assessed before and immediately after five min of thigh‐cuff occlusion. For this measurement, participants rested in the prone position with their legs supported with cushions under their ankles. During session 3, CEU imaging was conducted at rest and immediately after the 5‐min bout of intermittent isometric plantar‐flexion exercise with participants in the seated position. Image and acoustic intensity (aU) data were obtained from the belly of the medial gastrocnemius at the point of the greatest leg circumference, with the ultrasound transducer secured to the leg using a foam probe holder.

Continuous harmonic power‐Doppler imaging (Philips Diagnostic Ultrasound Systems model iE33, Philips Medical Systems) was performed with a linear‐array transducer (Philips L9‐3), using a low mechanical index (0.10), 87% gain, and 5 cm depth. All ultrasound settings were held constant within and between participants. Contrast solution consisting of 1.5 ml of lipid shelled octafluoropropane microbubbles (Definity, Bristol‐Myers Squibb Medical Imaging) mixed to 50 ml with saline was infused intravenously (antecubital fossa) at a constant rate of 200 ml/hour, using a syringe pump (Alaris PK) that was continuously rocked using a custom‐built mixing platform at approximately 20 rpm to prevent agent sedimentation. Each CEU measurement required a total of three min of contrast infusion, with the first two min of infusion required to achieve a steady‐state concentration of microbubbles. This was followed by a pulse of high‐energy ultrasound (mechanical index = 1.07) for microbubble destruction, and at least 50 s (range 50–60 s) of image acquisition to assess the kinetics of microbubble replenishment.

#### 2.7.1 Microvascular image analysis

QLAB software (Philips Healthcare) was used to generate time‐intensity curves for analysis of the contrast (image‐intensity) replenishment kinetics. An examiner (A.L.M.) manually selected a representative quadrilateral region of interest (903 ± 140 mm^2^); and the selected area was automatically transposed for repeated measurements. Time‐intensity data were exported for background subtraction (Excel, version 15.0 Microsoft Corporation, 2013), where the background intensity was set at 0.98 s (for resting data) or 0.49 s (for cuff occlusion and exercise data) from the moment of bubble‐destruction to exclude the contributions of the faster‐filling, larger non‐capillary vessels from the measured responses (Sacre et al., 2015). The area under the curve was calculated as the total accumulated change in acoustic intensity during the first 50 s post‐cuff occlusion or post‐exercise. Using a 3‐s moving average, we identified the time‐to‐peak acoustic intensity as the duration from microbubble destruction until its peak response (i.e., plateau acoustic intensity).

Time and acoustic intensity data for the same period, that is, from microbubble destruction until peak intensity, were used for curve fitting and the determination of microvascular parameters. Data were fitted to an exponential function *y = A[1−exp^−βt^]* for the analysis of the time‐intensity replenishment kinetics, where: y is the acoustic intensity at time t (in seconds); A is the peak acoustic intensity, which gives an estimation of microvascular blood volume; and β is the rate of appearance of the microbubbles (i.e., the rate constant), which estimates microvascular flow velocity (Wei et al., 1998). Skeletal muscle microvascular blood flow (perfusion) was calculated as the product of blood volume and velocity (A*β). Curve fitting was performed using SigmaPlot software version 13.0 (Systat Software, Inc). The goodness of fit for the CEU data, based on the adjusted *R* ^2^, was 0.93 ± 0.08.

### 2.8 Statistical analysis

Exercise‐induced skeletal muscle microvascular perfusion was the primary outcome of interest and therefore a priori sample size calculations were performed based on the mean of previously reported differences between young and older participants (effect size 1.4–1.96; Durham et al., 2010; Hildebrandt et al., 2017). Assuming a power of 90% and an alpha level of 0.05, a conservative sample size of 12 participants in each group was required to detect expected differences between young and older adults.

The Gaussian distribution and homogeneity of variance of the data were confirmed using Shapiro Wilk and Levene tests. Baseline comparisons were performed using independent sample *t* and chi‐square tests. A natural logarithm (Log_n_) transformation was effective in normalizing the non‐normal variables (post‐occlusion muscle microvascular blood volume, and post‐exercise muscle microvascular perfusion). Whole‐leg blood flow and microvascular perfusion responses to cuff occlusion and exercise were analyzed using a 2‐way (group × time) mixed analysis of variance (ANOVA), followed by Tukey's post hoc test when there were significant main effects or interactions. Correlations between leg blood flow and microvascular perfusion parameters were assessed using Pearson's or Spearman's correlation coefficient. *p* < .05 was considered statistically significant. Statistical analyses were performed using SPSS 22.0 for Windows software (SPSS Inc). Data are presented as mean and standard deviation (*SD*) unless otherwise stated. Within‐day test–retest reliability was assessed using the intra‐class correlation coefficient (ICC), and classified according to Fleiss (1999) as >0.75 excellent reliability, 0.40–0.75 good reliability, and <0.40 poor reliability. The coefficient of variation for each individual was calculated as the ratio of the standard deviation to the mean (CV%).

## 3 RESULTS

### 3.1 Participant characteristics

Participant characteristics are presented in Table 1. As stated previously, data presented for the older participant group, except the reliability data, have previously been published (Meneses et al., 2018). Young (26 ± 3 years) and older (68 ± 7 years) groups were well‐matched for the number of males (*n* = 6) and females (*n* = 6). Older participants had significantly (*p* < .05) higher body‐mass index, low‐density lipoprotein cholesterol, total cholesterol, and resting mean arterial pressure. Two (17%) participants in the older groups were taking prescribed anti‐hypertensive medications (angiotensin‐converting enzyme [ACE] inhibitor and calcium channel blocker), two were taking aspirin, and two were taking statins. Two female participants in the young group were taking contraceptive hormones (Ethinylestradiol). The self‐reported habitual physical activity levels were not significantly different between groups (Young: 3.9 ± 1.8 hr/week; Older: 2.8 ± 2.2 hr/week; *p* = .17).

**TABLE 1:** Participant characteristics

Parameter	Young (*n* = 12)	Older (*n* = 12)	*p* value
Age, years	26 ± 3	68 ± 7	<.01
Male sex, *n* (%)	6 (50)	6 (50)	.68
Weight, kg	72.8 ± 11.5	79.7 ± 12.0	.13
Height, cm	174.3 ± 8.6	172.3 ± 7.0	.52
Body mass index, kg/m^2^	23.9 ± 2.4	26.9 ± 4.0	.03
ECMM, kg	1.87 ± 0.36	1.98 ± 0.22	.33
Plantar‐flexion MVC force, *N*	1,385 ± 384	908 ± 336	<.01
Plantar‐flexion MVC force/ECMM, *N*/kg	741 ± 154	455 ± 154	<.01
Resting hemodynamics			
Systolic blood pressure, mm Hg	117 ± 11	136 ± 18	<.01
Diastolic blood pressure, mm Hg	66 ± 7	77 ± 7	<.01
Mean arterial blood pressure, mm Hg	79 ± 8	97 ± 11	<.01
Heart rate, beats/min	57 ± 10	61 ± 9	.39
Resting ankle‐brachial index	1.16 ± 0.13	1.16 ± 0.12	.93
Blood biochemistry			
Hemoglobin, mmol/L	14.39 ± 1.42	13.71 ± 0.7	.14
Hematocrit, %	42.5 ± 3.9	40.3 ± 1.7	.08
Fasting glucose, mmol/L	4.86 ± 0.46	5.16 ± 0.37	.08
LDL cholesterol, mmol/L	2.88 ± 0.72	3.62 ± 0.65	.01
HDL cholesterol, mmol/L	1.52 ± 0.25	1.53 ± 0.45	.97
Total cholesterol, mmol/L	4.76 ± 0.75	5.73 ± 0.74	<.01
Triglycerides, mmol/L	0.79 ± 0.28	1.33 ± 0.94	.06

Note

Values are mean ± *SD* or number (frequency distribution [%]). Data presented for the older participant group have previously been published (Meneses et al., 2018).

Abbreviations: ECMM, estimated calf muscle mass; HDL, high‐density lipoprotein; LDL, low‐density lipoprotein; MVC, maximum voluntary contraction.

Plantar‐flexion MVC force was lower in older participants compared with the young participants and remained lower when adjusted for calf muscle mass (Table 1). As such, the target force of 400 N during the 5‐min plantar‐flexion exercise tests reflected a higher relative intensity in the older participants (63 ± 22% MVC) compared with young participants (39 ± 11% MVC; *p* < .01).

### 3.2 Post‐occlusion whole‐leg blood flow and muscle microvascular perfusion

**FIGURE 1:**
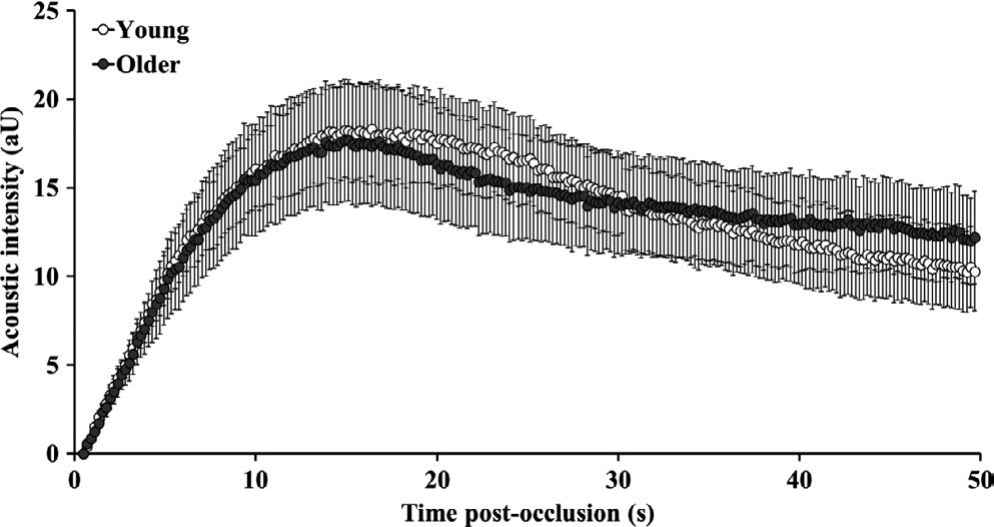
Post‐occlusion CEU time‐intensity curves in young (open circles) and older (shaded circles) participants. Data points represent group mean and error bars are *SEM*. Data presented for the older participant group have previously been published (Meneses et al., 2018)

**FIGURE 2:**
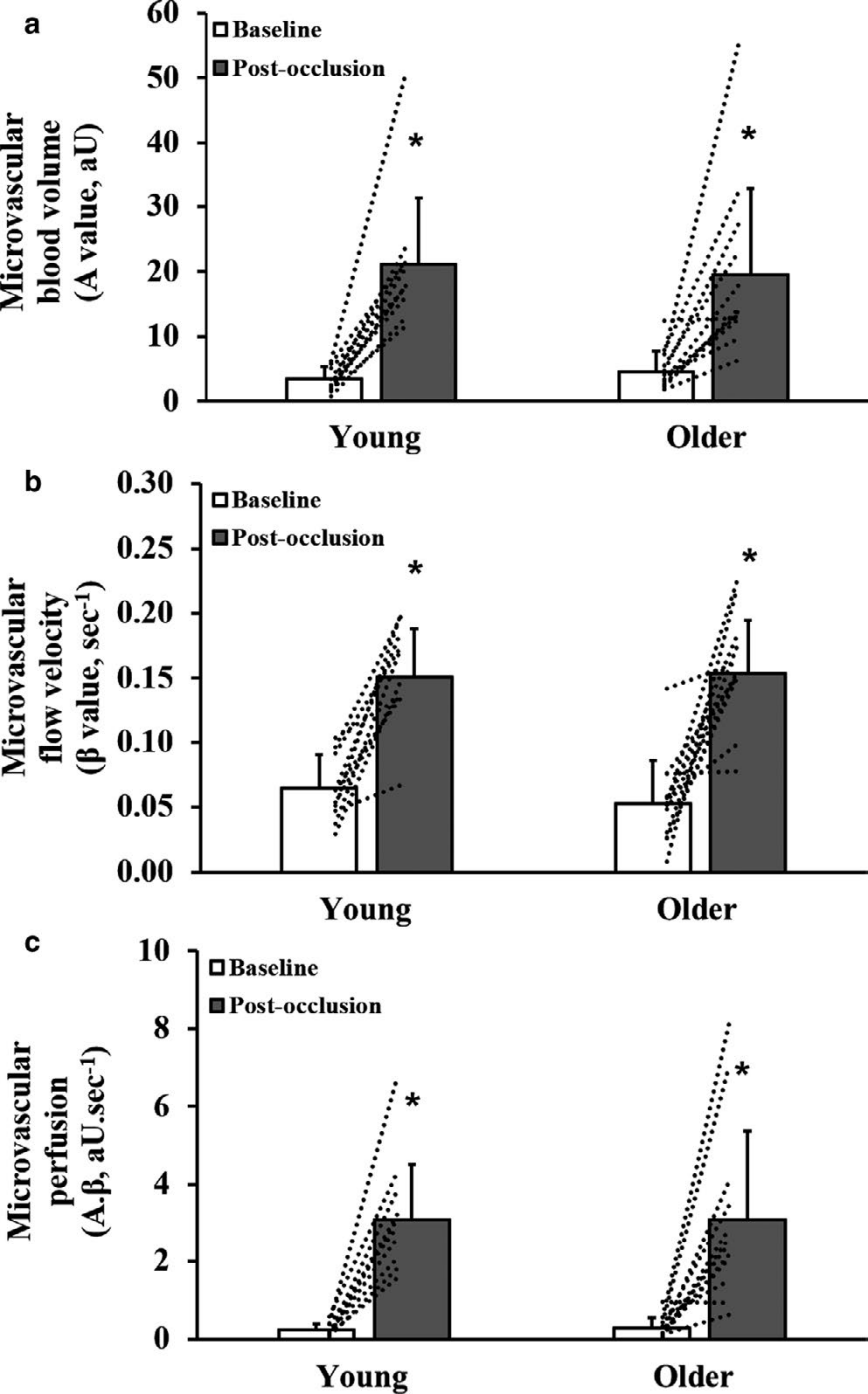
Calf muscle microvascular blood volume (panel a), flow velocity (panel b), and perfusion (panel c) at baseline and following cuff occlusion in young and older participants. Values are mean and *SD*. Dotted lines indicate individual participant responses. *Significantly different from baseline (*p* < .05). Data presented for the older participant group have previously been published (Meneses et al., 2018)

Whole‐leg blood flow and vascular conductance responses were not different between the age groups at rest or following cuff occlusion (Table 2). The mean post‐occlusion CEU time‐intensity microvascular perfusion curves in young and older participants are shown in Figure 1. Time‐to‐peak acoustic intensity was not different between groups (Young: 18.62 ± 3.77; Older: 17.79 ± 3.98 s; *p* = .60). The total area under the curve was also not different between groups (Young: 676 ± 359; Older: 659 ± 452 aU.s; *p* = .41). Calf muscle microvascular perfusion parameters, at baseline and post‐occlusion, are shown in Figure 2. Baseline (pre‐occlusion) muscle microvascular blood volume, flow velocity, and perfusion were not different between groups (*p* > .05). In comparison with baseline, microvascular blood volume (Figure 2a), flow velocity (Figure 2b), and perfusion (Figure 2c) increased in both groups during post‐occlusion reactive hyperemia (*p* < .001), with no significant differences between young and older participants (*p* > .05).

**TABLE 2:** Whole‐leg blood flow and leg vascular conductance

Parameter	Young (*n* = 12)	Older (*n* = 12)	*p* value
Post‐occlusion measures (supine)			
Baseline blood flow, ml.100 ml^−1^.min^−1^	2.18 ± 0.94	1.94 ± 0.65	.44
Baseline vascular conductance, ml.100 ml^−1^.min^−1^.mm Hg^−1^	0.03 ± 0.01	0.02 ± 0.01	.06
Post‐occlusion blood flow, ml.100 ml^−1^.min^−1^	17.51 ± 7.48	16.55 ± 6.02	.71
Post‐occlusion vascular conductance, ml.100 ml^−1^.min^−1^.mm Hg^−1^	0.22 ± 0.08	0.18 ± 0.07	.19
Exercise measures (seated)			
Baseline blood flow, ml.100 ml^−1^.min^−1^	1.12 ± 0.31	1.73 ± 0.95	.07
Baseline vascular conductance, ml.100 ml^−1^.min^−1^.mm Hg^−1^	0.010 ± 0.003	0.018 ± 0.013	.14
Exercise blood flow (plateau), ml.100 ml^−1^.min^−1^	31.19 ± 9.21	33.12 ± 8.55	.61
Exercise vascular conductance (plateau), ml.100 ml^−1^.min^−1^.mm Hg^−1^	0.323 ± 0.114	0.271 ± 0.060	.12
Exercise mean arterial pressure, mm Hg	99 ± 12	117 ± 22	.04
Exercise heart rate, beats/min	72 ± 12	68 ± 13	.47

Note

Values are mean ± *SD*. Data presented for the older participant group have previously been published (Meneses et al., 2018).

### 3.3 Exercise whole‐leg blood flow

**FIGURE 3:**
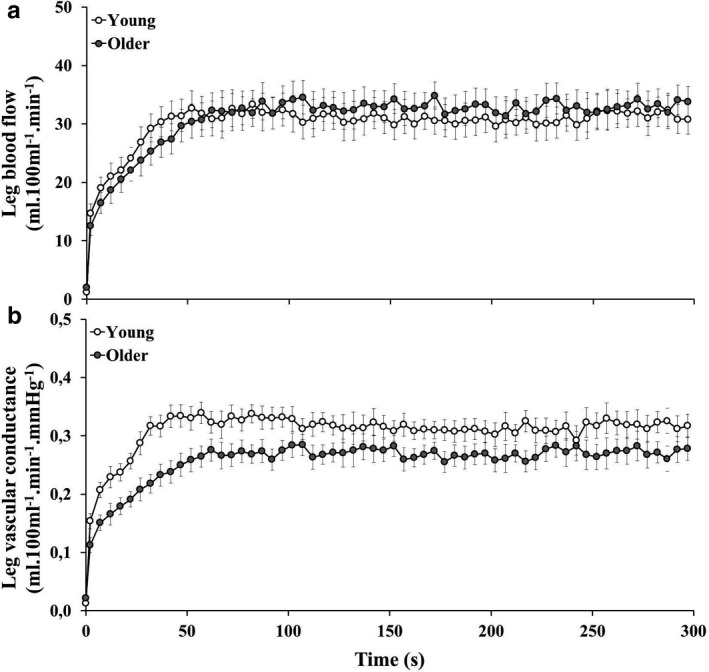
Whole‐leg blood flow (panel a) and vascular conductance (panel b) during plantar‐flexion exercise in young (open circles) and older (shaded circles) participants. Data points represent group mean and error bars are *SEM*. Data presented for the older participant group have previously been published (Meneses et al., 2018)

The increase in heart rate (Young: ∆14 ± 5 bpm; Older: ∆8 ± 8 bpm; *p* = .05) and mean arterial pressure (Young: ∆20 ± 11; Older: ∆26 ± 27 mmHg; *p* = .13) from rest to exercise were not significantly different between groups. Contraction‐by‐contraction whole‐leg blood flow and vascular conductance during plantar‐flexion exercise are illustrated in Figure 3a,b, respectively, and parameters are described in Table 2. Baseline (seated rest) whole‐leg blood flow was not different between groups (*p* = .07). The magnitude of whole‐leg blood flow (*p* = .61) and vascular conductance (*p* = .12) during exercise was not different between groups.

### 3.4 Post‐exercise muscle microvascular perfusion

**FIGURE 4:**
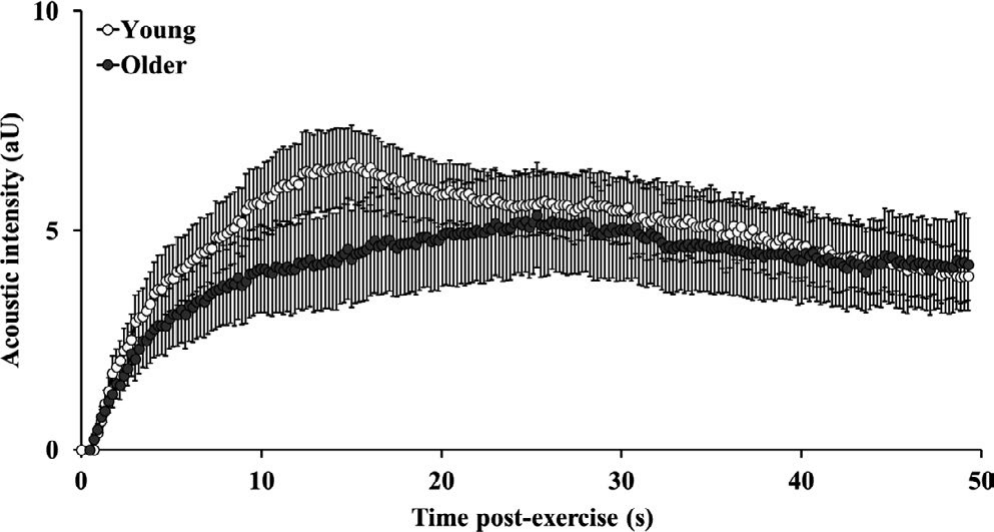
Post‐exercise CEU time‐intensity curves in young (open circles) and older (shaded circles) participants. Data points represent group mean and error bars are *SEM*. Data presented for the older participant group have previously been published (Meneses et al., 2018)

**FIGURE 5:**
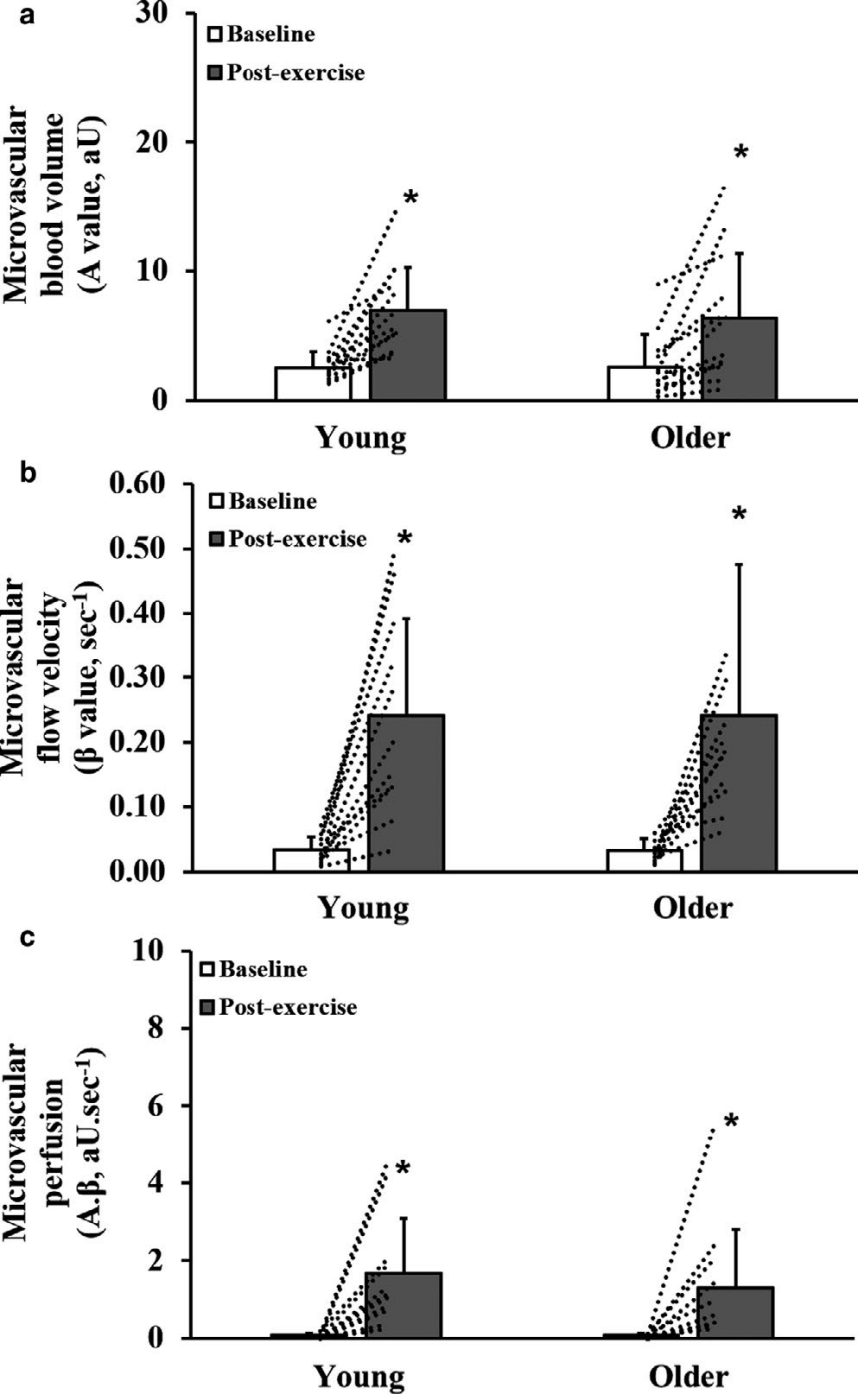
Calf muscle microvascular blood volume (panel a), flow velocity (panel b), and perfusion (panel c) before and following plantar‐flexion exercise in young and older participants. Values are mean and *SD*. Dotted lines indicate individual participant responses. *Significantly different from baseline (*p* < .05). Data presented for the older participant group have previously been published (Meneses et al., 2018)

The mean CEU time‐intensity curves for the period immediately following plantar‐flexion exercise in young and older participants are shown in Figure 4. Time‐to‐peak acoustic intensity was longer in the older (30.35 ± 14.46 s) than in the young participants (16.83 ± 3.22 s; *p* < .01). The total area under the curve was not different between groups (Young: 269 ± 124; Older: 264 ± 224 aU.s; *p* = .94). Calf muscle microvascular perfusion parameters, before and after exercise, are shown in Figure 5. Baseline (pre‐exercise) muscle microvascular blood volume, flow velocity, and perfusion were not different between groups (*p* > .05). Microvascular blood volume (Figure 5a), flow velocity (Figure 5b), and perfusion (Figure 5c) increased with exercise in both young and older groups (*p* < .001), with no significant differences between groups (*p* > .05).

### 3.5 Relationships between leg blood flow and microvascular perfusion

Given the lack of differences between groups for muscle microvascular perfusion parameters, data from the young and older participants were combined for correlations. A higher post‐occlusion whole‐leg blood flow and vascular conductance were significantly related to a shorter post‐occlusion time‐to‐peak CEU acoustic intensity (*n* = 24; *r* = −.53; *p* = .01 and *r* = −.43; *p* = .04, respectively). Post‐occlusion whole‐leg blood flow and vascular conductance were not significantly correlated with calf muscle microvascular perfusion (*n* = 24; *r* = .39; *p* = .07 and *r* = .42; *p* = .05, respectively). There were no significant correlations between exercise whole‐leg blood flow and post‐exercise CEU microvascular parameters, except for post‐exercise TTP that was negatively correlated with exercise leg vascular conductance (*n* = 24; *r* = −.47; *p* = .03).

### 3.6 Reliability of CEU muscle microvascular perfusion parameters

The mean test–retest data, ICC, and coefficient of the variation of CEU skeletal muscle microvascular perfusion parameters at rest, post‐occlusion, and post‐exercise are presented in Table 3. Resting supine (pre‐cuff occlusion) and seated (pre‐exercise) muscle microvascular perfusion values indicate that there was no residual effect of prior cuff occlusion or exercise on repeated measures (*p* > .05). Under resting conditions, the ICC values indicated good to excellent reliability for muscle microvascular perfusion (ICC range: 0.73–0.96). Post‐occlusion CEU parameters showed excellent within‐day reliability with ICC values ranging from 0.82 to 0.96, and CV values from 13% to 39%. For the post‐exercise CEU parameters, the ICC values ranged from 0.91 to 0.98, and CV values from 7% to 22%.

**TABLE 3:** Reliability of muscle microvascular perfusion parameters measured with CEU

Parameter	Young		ICC	CV%	*p* value	Older		ICC	CV%	*p* value
	Test 1	Test 2				Test 1	Test 2			
Post‐occlusion measures (supine)										
Baseline microvascular perfusion (A.β, aU.s^−1^)	0.23 ± 0.16	0.26 ± 0.19	0.85	24	.51	0.39 ± 0.48	0.30 ± 0.31	0.92	30	.21
Post‐occlusion TTP (s)	18.96 ± 4.00	19.54 ± 3.74	0.91	7	.44	17.13 ± 4.05	18.51 ± 3.85	0.92	9	.08
Post‐occlusion AUC (aU.s)	724 ± 388	659 ± 411	0.98	9	.06	813 ± 566	678 ± 468	0.93	18	.20
Post‐occlusion microvascular perfusion (A.β, aU.s^−1^)	3.19 ± 1.35	3.09 ± 1.59	0.94	12	.67	3.51 ± 2.53	3.00 ± 2.44	0.96	22	.15
Exercise measures (seated)										
Baseline microvascular perfusion (A.β, aU.s^−1^)	0.09 ± 0.06	0.11 ± 0.08	0.73	31	.11	0.08 ± 0.05	0.09 ± 0.06	0.96	26	.62
Post‐exercise TTP intensity (s)	17.35 ± 4.13	16.54 ± 3.52	0.84	17	.84	27.98 ± 13.50	30.44 ± 14.01	0.94	30	.29
Post‐exercise AUC (aU.s)	257 ± 127	266 ± 111	0.86	13	.73	248 ± 207	288 ± 247	0.93	35	.93
Post‐exercise microvascular perfusion (A.β, aU.s^−1^)	2.14 ± 1.98	1.42 ± 0.90	0.82	28	.09	1.21 ± 1.37	1.35 ± 1.50	0.96	39	.43

Note

Values are mean ± *SD*.

Abbreviations: AUC, area under the curve; TTP, time‐to‐peak acoustic intensity.

## 4 DISCUSSION

This study aimed to compare the skeletal muscle microvascular perfusion and whole‐leg blood flow responses to standardized cuff occlusion and matched submaximal intensity exercise protocols between young and older participants. The secondary aim was to assess the test–retest reliability of CEU skeletal muscle perfusion parameters. We found that post‐occlusion and post‐exercise muscle microvascular perfusion responses were not different between age groups, and this was consistent with the whole‐leg blood flow responses, which were also similar between old and younger participants. Our findings also show that CEU imaging is a reliable technique for the assessment of calf muscle microvascular perfusion responses to cuff occlusion and leg exercise.

### 4.1 Post‐occlusion whole‐leg blood flow and calf muscle microvascular perfusion

Previous studies that have assessed the influence of age on muscle microvascular perfusion have often failed to account for any differences in whole‐leg arterial inflow. In the present study, post‐occlusion whole‐leg blood flow and vascular conductance were not different between young and older adults. Our findings are in line with previous studies (Hildebrandt et al., 2017; Schank et al., 2006) using strain‐gauge plethysmography, which reported no age‐related differences in post‐occlusion whole‐limb blood flow (reactive hyperemia) in recreationally active adults. This is also in line with a study showing no age‐related differences in lower limb flow‐mediated dilatation, induced by cuff‐occlusion, in both active and inactive young and older adults (Olive et al., 2002). Notably, several previous studies have reported age‐related impairments in endothelial function (flow‐mediated dilation) of the brachial artery (Black et al., 2009; Celermajer et al., 1994; Gates et al., 2007). However, it has been shown that vascular responsiveness is not uniform across upper and lower limbs (Donato et al., 2006; Thijssen et al., 2011; Wray et al., 2010). The assessment of vascular function in the leg, rather than the arm, may be more relevant when considering the impact of age and age‐related conditions, such as peripheral arterial disease, on exercise tolerance.

We found no differences in post‐occlusion calf muscle microvascular perfusion between young and older participants. Our findings differ from previous studies using CEU (Thomas et al., 2014) and magnetic resonance imaging (MRI; Kos et al., 2009; Schulte et al., 2008) that reported reduced post‐occlusion calf muscle perfusion in older adults. The discrepancy between study findings is not likely to be related to the provocation stimuli, as these prior studies showed an impaired response in the older adults using varied cuff occlusion protocols (1–6 min occlusion at supra‐systolic pressures). The variance in age between young and older participants, as well as the differences in body‐mass index, lipid profile, and blood pressure, which often correlate with alterations in vascular function (Benjamin et al., 2004), were within similar ranges in the current and prior studies. Nonetheless, unlike prior observations (Thomas et al., 2014), the microbubble replenishment curves, and the microvascular perfusion responses following cuff‐occlusion were almost identical between the young and older participants in the current study. As post‐occlusion reactive hyperemia reflects vasodilatory capacity and is influenced by alterations in vascular resistance in the (macro and micro) vascular network (Sandoo et al., 2010), our data suggest that the capacity of the lower limb vascular system is well‐preserved in healthy older adults.

### 4.2 Whole‐leg blood flow and vascular conductance during exercise

Our findings of similar whole‐leg blood flow and vascular conductance during exercise in young and older adults are consistent with findings recently reported in recreationally active older and young adults during incremental intermittent plantar‐flexion exercise (Reilly et al., 2018). In this prior study, participants were stratified according to sex, but no sex‐specific effects in either leg blood flow or vascular conductance responses during exercise were observed. Conversely, leg hemodynamic responses during unilateral knee extension exercise were similar between older and young recreationally active males, but attenuated in older compared with young women (Parker et al., 2008). While there might be a sex‐specific effect on exercising limb hemodynamics with aging, our study sample was not powered for comparisons of subgroups according to sex.

The age‐related attenuation in exercising leg hemodynamics reported in previous studies has been partly attributed to the smaller leg muscle mass in older participants (Donato et al., 2006; Parker et al., 2008). Thus, when exercise responses were expressed per unit of muscle mass, leg blood flow, and vascular conductance responses were not significantly different between the young and older groups (Donato et al., 2006; Parker et al., 2008). Estimated calf muscle mass was not different between the young and older participants in this study. Furthermore, leg blood flow was assessed relative to the volume of tissue per minute (i.e., ml.100 ml^−1^.min^−1^). Thus, our results are not likely to be affected by differences in muscle mass between young and older individuals. Differences in exercise workload employed in previous studies also likely explain the attenuated response in older compared with young participants (Donato et al., 2006). Importantly, we used the same absolute workload across both groups. While the relative intensity differed, we showed that total limb blood flow and vascular conductance, normalized for muscle mass, were not different between young and older participants. This approach is a strength of the current study which ensures that any variance in muscle microvascular blood flow between groups can be attributed to the age of the participants and is not likely influenced by variance in mechanical work (and therefore oxygen demand) and whole limb blood flow.

While the magnitude of the whole‐limb blood flow and vascular conductance response during exercise was not different between age groups, it is interesting to note that the immediate rise in whole‐limb vascular conductance during exercise appears to be slower in the older compared to the younger group. It is likely that this reflects a disruption of the immediate vasoregulatory response during exercise and is possibly related to increased vascular stiffness (Wen et al., 2015) or endothelial dysfunction (Parker et al., 2006) in older adults. Increased sympathetic tone in older adults may also play a role, although it was recently reported that age‐related differences in the vasodilation response to exercise are not fully explained by sympathetic vasoconstriction (Hughes et al., 2017). Further studies are needed to determine the kinetics of the blood flow response to exercise and to investigate the mechanisms of any age‐related effects.

### 4.3 Post‐exercise calf muscle microvascular perfusion

Consistent with our findings for whole‐leg blood flow, we also found no differences in post‐exercise calf muscle microvascular perfusion between young and older participants. Our results contrast with previous findings showing reduced peak microvascular perfusion of the soleus (Tonson et al., 2017) and vastus lateralis (Hildebrandt et al., 2017) muscles following a single submaximal isometric contraction. In line with our findings, these studies reported no age‐related differences in peak microvascular perfusion of the medial gastrocnemius (Tonson et al., 2017) and the vastus intermedius muscle (Hildebrandt et al., 2017). Therefore, the possibility that age‐related differences in muscle perfusion are muscle group‐specific requires further investigation.

Impaired muscle perfusion may not necessarily reflect altered muscle structure with advancing age, as the muscle microvascular responses reported by Hildebrandt et al. (2017) were seen in the absence of differences in muscle capillary density or fiber composition between young and middle‐age participants. Instead, age‐related differences in muscle microvascular hyperemia may reflect differences in vasodilatory capacity or responsiveness. In line with Hildebrandt et al. (2017), older participants in our study demonstrated a slower pattern of reperfusion immediately after exercise compared with young adults. Indeed, we found a slower TTP response, although the AUC (i.e., total change in microvascular volume) was not significantly different between young and older participants. This altered response suggests there may be an attenuated vascular reactivity in the lower limbs of older adults, although this appeared to have no measurable effect on the perfusion response following exercise.

### 4.4 Reliability of muscle microvascular perfusion parameters

The current study demonstrates good to excellent within‐day reliability for measures of resting perfusion parameters, and excellent reliability of post‐occlusion and post‐exercise perfusion parameters in young and older adults (ICC range 0.82–0.98). In contrast, Thomas et al. (2014) reported poor reliability of post‐occlusion (60 s at 200 mm Hg cuff pressure) calf muscle CEU parameters in both young and older participants. While in this prior study TTP was the most reliable parameter (ICC 0.77–0.78), other parameters such as peak acoustic intensity and area under the curve demonstrated wide variance across both groups (ICC range 0.01–0.89; CV range 23%–87%). In the current study, CEU data were fitted to biokinetic models to remove noise (Tang et al., 2011) and estimate the microvascular volume and flow velocity for the determination of microvascular blood flow. Using a similar CEU technique to that employed in the present study, albeit using an incremental (5 s bins) rather than continuous real‐time imaging protocol, Lindner et al. (2008) demonstrated a variability of 20% for resting values and 15% for maximal exercise values in healthy middle‐aged participants (*n* = 5; median 47 years, range 41–52 years). This is similar to the CV% values reported in the current study (24%–30% and 7%–22% for resting and post‐exercise parameters, respectively).

Technical factors influencing the reliability of CEU measurements include the CEU ultrasound and software settings (i.e., mechanical index, focal depth, dynamic range, gain, pulse frequency) and contrast agent administration (i.e., infusion and handling; Tang et al., 2011), which were standardized for all participants and tests in the current study. We examined a larger area of muscle tissue (i.e., region of interest) compared to Thomas et al. (2014) (903 ± 140 mm^2^ vs. 158 ± 19 mm^2^, respectively), which may have contributed to the improved reliability reported in our study. The region of interest represents the area over which the CEU data are captured to generate the time‐intensity curve; thus, a larger region of interest includes more data and minimizes variability, as previously demonstrated in the myocardium (Fornwalt et al., 2009). An important technical factor is that CEU imaging is susceptible to movement artifacts (Greis, 2011; Weber et al., 2007), and therefore imaging acquisition in the current study was performed in the period immediately after the completion of exercise (post‐contraction). In the study by Lindner et al. (2008), image acquisition commenced during exercise and continued during the recovery period, which may have resulted in alterations in signal intensity and contributed to the reported measurement variance. Microvascular perfusion can be normalized to the blood pool concentration of the contrast agent (Davidson et al., 2016; Shim et al., 2014), which might further reduce variability in CEU parameters. Current CEU studies have not commonly used this adjustment, and the reproducibility of this approach needs to be tested in future studies. Using our standardized stimulus (cuff occlusion and exercise), contrast administration, imaging, and image‐analysis procedures, we have demonstrated that CEU muscle perfusion parameters are highly reproducible in both young and older adults.

### 4.5 Limitations

While the study was powered to detect age‐group differences in post‐exercise microvascular perfusion, the study sample was small and did not permit the comparison of sex‐differences and other factors that may influence the findings. In this study, the older and younger groups were well‐matched for total physical activity levels and none of the participants reported participating in supervised exercise training. We did not assess the intensity of habitual physical activity, and we suggest that future studies consider a more thorough assessment of physical activity to account for any impact it may have on age‐related comparisons. It was not possible to make simultaneous measurements of whole‐leg blood flow and calf muscle microvascular perfusion. Synchronized measures would have strengthened our ability to assess the association between whole‐leg blood flow and microvascular perfusion in response to exercise. Our experiments were all performed at the same time of day to mitigate the effect of circadian changes in cardiovascular control. We acknowledge that within‐day reliability measures may yield different results compared with those in which repeated tests are performed across longer time intervals (i.e., days or weeks). Therefore, we suggest that future studies assess the between‐day reliability of CEU measures using the same standardized data acquisition and processing protocols. This is particularly important for establishing the utility of CEU for the assessment of longitudinal changes in muscle microvascular perfusion that might occur with aging, disease progression, or in response to treatment interventions.

## 5 CONCLUSION

Using standardized contrast administration, imaging, and image‐analysis procedures, this study demonstrates that contrast‐enhanced ultrasound imaging is a reliable method for the assessment of muscle microvascular perfusion parameters. Using these methods, this study has established that the calf muscle microvascular perfusion responses to cuff‐occlusion and matched‐intensity leg exercise are not different between healthy young and older adults. These findings suggest that muscle microvascular impairments associated with age‐related conditions such as type 2 diabetes, peripheral arterial disease, and heart failure are more likely the result of disease‐related pathology than a function of aging per se.

## CONFLICT OF INTEREST

No conflicts of interest, financial or otherwise, are declared by the authors.

## AUTHOR CONTRIBUTIONS

Conception and design of the study (ALM, TGB, KG, CDA); data collection (ALM, MCYN); analyses and interpretation of data (ALM, MCYN, TGB, RM, JG, YH, MK, KG, CDA); writing/critical revision of the manuscript for important intellectual content (ALM, MCYN, TGB, RM, JG, YH, MK, KG, CDA).

## References

[phy214580-bib-0001] Amarteifio, E. , Wormsbecher, S. , Krix, M. , Demirel, S. , Braun, S. , Delorme, S. , Bockler, D. , Kauczor, H. U. , & Weber, M. A. (2012). Dynamic contrast‐enhanced ultrasound and transient arterial occlusion for quantification of arterial perfusion reserve in peripheral arterial disease. European Journal of Radiology, 81, 3332–3338. 10.1016/j.ejrad.2011.12.030 22285606

[phy214580-bib-0002] Askew, C. D. , Green, S. , Hou, X. Y. , & Walker, P. J. (2002). Physiological and symptomatic responses to cycling and walking in intermittent claudication. Clinical Physiology and Functional Imaging, 22, 348–355. 10.1046/j.1475-097X.2002.00442.x 12487008

[phy214580-bib-0003] Askew, C. D. , & Matthews, J. (2012). Measurement of leg blood flow during exercise using strain gauge plethysmography: Effect of venous occlusion pressure In: 59th Annual Meeting of the American College of Sports Medicine (ACSM) (pp. 959–1065). Medicine and Science in Sports and Exercise.

[phy214580-bib-0004] Australian Bureau of Statistics . (2011/2012). National nutrition and physical activity survey.

[phy214580-bib-0005] Benjamin, E. J. , Larson, M. G. , Keyes, M. J. , Mitchell, G. F. , Vasan, R. S. , Keaney, J. F. Jr , Lehman, B. T. , Fan, S. , Osypiuk, E. , & Vita, J. A. (2004). Clinical correlates and heritability of flow‐mediated dilation in the community: The Framingham Heart Study. Circulation, 109, 613–619. 14769683 10.1161/01.CIR.0000112565.60887.1E

[phy214580-bib-0006] Bigler, M. , Koutsantonis, D. , Odriozola, A. , Halm, S. , Tschanz, S. A. , Zakrzewicz, A. , Weichert, A. , & Baum, O. (2016). Morphometry of skeletal muscle capillaries: The relationship between capillary ultrastructure and ageing in humans. Acta Physiologica, 218, 98–111. 27174490 10.1111/apha.12709

[phy214580-bib-0007] Black, M. A. , Cable, N. T. , Thijssen, D. H. , & Green, D. J. (2009). Impact of age, sex, and exercise on brachial artery flow‐mediated dilatation. American Journal of Physiology Heart and Circulatory Physiology, 297, H1109–1116. 10.1152/ajpheart.00226.2009 19633208 PMC2755978

[phy214580-bib-0008] Celermajer, D. S. , Sorensen, K. E. , Spiegelhalter, D. J. , Georgakopoulos, D. , Robinson, J. , & Deanfield, J. E. (1994). Aging is associated with endothelial dysfunction in healthy men years before the age‐related decline in women. Journal of the American College of Cardiology, 24, 471–476. 10.1016/0735-1097(94)90305-0 8034885

[phy214580-bib-0009] Chilibeck, P. D. , Paterson, D. H. , Cunningham, D. A. , Taylor, A. W. , & Noble, E. G. (1997). Muscle capillarization O2 diffusion distance, and VO2 kinetics in old and young individuals. Journal of Applied Physiology (1985), 82, 63–69. 10.1152/jappl.1997.82.1.63 9029199

[phy214580-bib-0010] Clarys, J. P. , & Marfell‐Jones, M. J. (1986). Anatomical segmentation in humans and the prediction of segmental masses from intra‐segmental anthropometry. Human Biology, 58, 771–782. 3804298

[phy214580-bib-0011] Coggan, A. R. , Spina, R. J. , King, D. S. , Rogers, M. A. , Brown, M. , Nemeth, P. M. , & Holloszy, J. O. (1992). Histochemical and enzymatic comparison of the gastrocnemius muscle of young and elderly men and women. Journal of Gerontology, 47, B71–B76. 1573181 10.1093/geronj/47.3.b71

[phy214580-bib-0012] Copp, S. W. , Hirai, D. M. , Ferreira, L. F. , Poole, D. C. , & Musch, T. I. (2010). Progressive chronic heart failure slows the recovery of microvascular O2 pressures after contractions in the rat spinotrapezius muscle. American Journal of Physiology Heart and Circulatory Physiology, 299, H1755–H1761. 20817826 10.1152/ajpheart.00590.2010 PMC3006296

[phy214580-bib-0013] Davidson, B. P. , Belcik, J. T. , Landry, G. , Linden, J. , & Lindner, J. R. (2017). Exercise versus vasodilator stress limb perfusion imaging for the assessment of peripheral artery disease. Echocardiography, 34, 1187–1194. 28664576 10.1111/echo.13601 PMC5568936

[phy214580-bib-0014] Davidson, B. P. , Belcik, J. T. , Mott, B. H. , Landry, G. , & Lindner, J. R. (2016). Quantification of residual limb skeletal muscle perfusion with contrast‐enhanced ultrasound during application of a focal junctional tourniquet. Journal of Vascular Surgery, 63, 148–153. 25065582 10.1016/j.jvs.2014.06.107 PMC4344419

[phy214580-bib-0015] Delp, M. D. , Behnke, B. J. , Spier, S. A. , Wu, G. , & Muller‐Delp, J. M. (2008). Ageing diminishes endothelium‐dependent vasodilatation and tetrahydrobiopterin content in rat skeletal muscle arterioles. The Journal of Physiology, 586, 1161–1168. 18063659 10.1113/jphysiol.2007.147686 PMC2375630

[phy214580-bib-0016] Donato, A. J. , Uberoi, A. , Wray, D. W. , Nishiyama, S. , Lawrenson, L. , & Richardson, R. S. (2006). Differential effects of aging on limb blood flow in humans. American Journal of Physiology Heart and Circulatory Physiology, 290, H272–H278. 16183733 10.1152/ajpheart.00405.2005

[phy214580-bib-0017] Durham, W. J. , Casperson, S. L. , Dillon, E. L. , Keske, M. A. , Paddon‐Jones, D. , Sanford, A. P. , Hickner, R. C. , Grady, J. J. , & Sheffield‐Moore, M. (2010). Age‐related anabolic resistance after endurance‐type exercise in healthy humans. FASEB Journal: Official Publication of the Federation of American Societies for Experimental Biology, 24, 4117–4127. 20547663 10.1096/fj.09-150177 PMC2996901

[phy214580-bib-0018] Egana, M. , & Green, S. (2005). Effect of body tilt on calf muscle performance and blood flow in humans. Journal of Applied Physiology (1985), 98, 2249–2258. 10.1152/japplphysiol.01235.2004 15661836

[phy214580-bib-0019] Fleiss, J. L. (1999). Reliability of measurement. The design and analysis of clinical experiments. John Wiley & Sons Inc.

[phy214580-bib-0020] Fornwalt, B. K. , Sprague, W. W. , Carew, J. D. , Merlino, J. D. , Fyfe, D. A. , Leon, A. R. , & Oshinski, J. N. (2009). Variability in tissue Doppler echocardiographic measures of dyssynchrony is reduced with use of a larger region of interest. Journal of the American Society of Echocardiography, 22, 478–485. 19450742 10.1016/j.echo.2009.02.019 PMC2804862

[phy214580-bib-0021] Frontera, W. R. , Hughes, V. A. , Fielding, R. A. , Fiatarone, M. A. , Evans, W. J. , & Roubenoff, R. (2000). Aging of skeletal muscle: A 12‐yr longitudinal study. Journal of Applied Physiology (1985), 88, 1321–1326. 10.1152/jappl.2000.88.4.1321 10749826

[phy214580-bib-0022] Gates, P. E. , Boucher, M. L. , Silver, A. E. , Monahan, K. D. , & Seals, D. R. (2007). Impaired flow‐mediated dilation with age is not explained by L‐arginine bioavailability or endothelial asymmetric dimethylarginine protein expression. Journal of Applied Physiology (1985), 102, 63–71. 10.1152/japplphysiol.00660.2006 16946027

[phy214580-bib-0023] Green, S. , Thorp, R. , Reeder, E. J. , Donnelly, J. , & Fordy, G. (2011). Venous occlusion plethysmography versus Doppler ultrasound in the assessment of leg blood flow during calf exercise. European Journal of Applied Physiology, 111, 1889–1900. 21234593 10.1007/s00421-010-1819-6

[phy214580-bib-0024] Greis, C. (2011). Quantitative evaluation of microvascular blood flow by contrast‐enhanced ultrasound (CEUS). Clinical Hemorheology and Microcirculation, 49, 137–149. 22214685 10.3233/CH-2011-1464

[phy214580-bib-0025] Groen, B. B. , Hamer, H. M. , Snijders, T. , van Kranenburg, J. , Frijns, D. , Vink, H. , & van Loon, L. J. (2014). Skeletal muscle capillary density and microvascular function are compromised with aging and type 2 diabetes. Journal of Applied Physiology (1985), 116, 998–1005. 10.1152/japplphysiol.00919.2013 24577061

[phy214580-bib-0026] Hildebrandt, W. , Schwarzbach, H. , Pardun, A. , Hannemann, L. , Bogs, B. , Konig, A. M. , Mahnken, A. H. , Hildebrandt, O. , Koehler, U. , & Kinscherf, R. (2017). Age‐related differences in skeletal muscle microvascular response to exercise as detected by contrast‐enhanced ultrasound (CEUS). PLoS One, 12, e0172771. 28273102 10.1371/journal.pone.0172771 PMC5342194

[phy214580-bib-0027] Hokanson, D. E. , Sumner, D. S. , & Strandness, D. E. Jr (1975). An electrically calibrated plethysmograph for direct measurement of limb blood flow. IEEE Transactions on Bio‐medical Engineering, 22, 25–29. 1110089 10.1109/tbme.1975.324535

[phy214580-bib-0028] Hughes, W. E. , Kruse, N. T. , & Casey, D. P. (2017). Sympathetic nervous system activation reduces contraction‐induced rapid vasodilation in the leg of humans independent of age. Journal of Applied Physiology (1985), 123, 106–115. 10.1152/japplphysiol.00005.2017 PMC6157482 28385914

[phy214580-bib-0029] Kos, S. , Klarhofer, M. , Aschwanden, M. , Scheffler, K. , Jacob, A. L. , & Bilecen, D. (2009). Simultaneous dynamic blood oxygen level‐dependent magnetic resonance imaging of foot and calf muscles: Aging effects at ischemia and postocclusive hyperemia in healthy volunteers. Investigative Radiology, 44, 741–747. 19809343 10.1097/RLI.0b013e3181b248f9

[phy214580-bib-0030] Kundi, R. , Prior, S. J. , Addison, O. , Lu, M. , Ryan, A. S. , & Lal, B. K. (2017). Contrast‐enhanced ultrasound reveals exercise‐induced perfusion deficits in claudicants. Journal of Vascular and Endovascular Surgery, 2, 1–16. 10.21767/2573-4482.100041 PMC5501290 28691118

[phy214580-bib-0031] Kusters, Y. H. , & Barrett, E. J. (2016). Muscle microvasculature's structural and functional specializations facilitate muscle metabolism. American Journal of Physiology Endocrinology and Metabolism, 310, E379–E387. 10.1152/ajpendo.00443.2015 26714849 PMC4888529

[phy214580-bib-0032] Lindner, J. R. , Womack, L. , Barrett, E. J. , Weltman, J. , Price, W. , Harthun, N. L. , Kaul, S. , & Patrie, J. T. (2008). Limb stress‐rest perfusion imaging with contrast ultrasound for the assessment of peripheral arterial disease severity. JACC Cardiovascular Imaging, 1, 343–350. 10.1016/j.jcmg.2008.04.001 19356447 PMC2651026

[phy214580-bib-0033] McDonough, P. , Behnke, B. J. , Musch, T. I. , & Poole, D. C. (2004). Effects of chronic heart failure in rats on the recovery of microvascular PO2 after contractions in muscles of opposing fibre type. Experimental Physiology, 89, 473–485. 15131070 10.1113/expphysiol.2004.027367

[phy214580-bib-0034] Meneses, A. L. , Nam, M. C. Y. , Bailey, T. G. , Magee, R. , Golledge, J. , Hellsten, Y. , Keske, M. A. , Greaves, K. , & Askew, C. D. (2018). Leg blood flow and skeletal muscle microvascular perfusion responses to submaximal exercise in peripheral arterial disease. American Journal of Physiology Heart and Circulatory Physiology, 315, H1425–H1433. 10.1152/ajpheart.00232.2018 30095999

[phy214580-bib-0035] Murphy, E. , Rocha, J. , Gildea, N. , Green, S. , & Egana, M. (2018). Venous occlusion plethysmography vs. Doppler ultrasound in the assessment of leg blood flow kinetics during different intensities of calf exercise. European Journal of Applied Physiology, 118, 249–260. 10.1007/s00421-017-3765-z 29192355

[phy214580-bib-0036] Olive, J. L. , DeVan, A. E. , & McCully, K. K. (2002). The effects of aging and activity on muscle blood flow. Dynamic Medicine, 1, 2. 12605712 10.1186/1476-5918-1-2 PMC150384

[phy214580-bib-0037] Parker, B. A. , Ridout, S. J. , & Proctor, D. N. (2006). Age and flow‐mediated dilation: A comparison of dilatory responsiveness in the brachial and popliteal arteries. American Journal of Physiology Heart and Circulatory Physiology, 291, H3043–H3049. 16861699 10.1152/ajpheart.00190.2006

[phy214580-bib-0038] Parker, B. A. , Smithmyer, S. L. , Pelberg, J. A. , Mishkin, A. D. , & Proctor, D. N. (2008). Sex‐specific influence of aging on exercising leg blood flow. Journal of Applied Physiology (1985), 104, 655–664. 10.1152/japplphysiol.01150.2007 18162481

[phy214580-bib-0039] Poole, D. C. , Copp, S. W. , Ferguson, S. K. , & Musch, T. I. (2013). Skeletal muscle capillary function: Contemporary observations and novel hypotheses. Experimental Physiology, 98, 1645–1658. 23995101 10.1113/expphysiol.2013.073874 PMC4251469

[phy214580-bib-0040] Reeder, E. J. , & Green, S. (2012). Dynamic response characteristics of hyperaemia in the human calf muscle: Effect of exercise intensity and relation to electromyographic activity. European Journal of Applied Physiology, 112, 3997–4013. 22441829 10.1007/s00421-012-2362-4

[phy214580-bib-0041] Reilly, H. , Lane, L. M. , & Egana, M. (2018). Lack of age‐specific influence on leg blood flow during incremental calf plantar‐flexion exercise in men and women. European Journal of Applied Physiology, 118, 989–1001. 29502172 10.1007/s00421-018-3833-z

[phy214580-bib-0042] Ryan, N. A. , Zwetsloot, K. A. , Westerkamp, L. M. , Hickner, R. C. , Pofahl, W. E. , & Gavin, T. P. (2006). Lower skeletal muscle capillarization and VEGF expression in aged vs. young men. Journal of Applied Physiology (1985), 100, 178–185. 10.1152/japplphysiol.00827.2005 16166239

[phy214580-bib-0043] Sacre, J. W. , Jellis, C. L. , Haluska, B. A. , Jenkins, C. , Coombes, J. S. , Marwick, T. H. , & Keske, M. A. (2015). Association of exercise intolerance in type 2 diabetes with skeletal muscle blood flow reserve. JACC Cardiovascular Imaging, 8, 913–921. 26189114 10.1016/j.jcmg.2014.12.033

[phy214580-bib-0044] Sandoo, A. , van Zanten, J. J. , Metsios, G. S. , Carroll, D. , & Kitas, G. D. (2010). The endothelium and its role in regulating vascular tone. The Open Cardiovascular Medicine Journal, 4, 302–312. 21339899 10.2174/1874192401004010302 PMC3040999

[phy214580-bib-0045] Saunders, N. R. , Pyke, K. E. , & Tschakovsky, M. E. (2005). Dynamic response characteristics of local muscle blood flow regulatory mechanisms in human forearm exercise. Journal of Applied Physiology (1985), 98, 1286–1296. 10.1152/japplphysiol.01118.2004 15579568

[phy214580-bib-0046] Scelsi, R. , Marchetti, C. , & Poggi, P. (1980). Histochemical and ultrastructural aspects of M. vastus lateralis in sedentary old people (age 65–89 years). Acta Neuropathologica, 51, 99–105. 7435151 10.1007/BF00690450

[phy214580-bib-0047] Schank, B. J. , Acree, L. S. , Longfors, J. , & Gardner, A. W. (2006). Differences in vascular reactivity between men and women. Angiology, 57, 702–708. 17235110 10.1177/0003319706295474

[phy214580-bib-0048] Schulte, A. C. , Aschwanden, M. , & Bilecen, D. (2008). Calf muscles at blood oxygen level‐dependent MR imaging: Aging effects at postocclusive reactive hyperemia. Radiology, 247, 482–489. 18372453 10.1148/radiol.2472070828

[phy214580-bib-0049] Shim, C. Y. , Kim, S. , Chadderdon, S. , Wu, M. , Qi, Y. , Xie, A. , Alkayed, N. J. , Davidson, B. P. , & Lindner, J. R. (2014). Epoxyeicosatrienoic acids mediate insulin‐mediated augmentation in skeletal muscle perfusion and blood volume. American Journal of Physiology. Endocrinology and Metabolism, 307, E1097–1104. 25336524 10.1152/ajpendo.00216.2014 PMC4269677

[phy214580-bib-0050] Tang, M. X. , Mulvana, H. , Gauthier, T. , Lim, A. K. , Cosgrove, D. O. , Eckersley, R. J. , & Stride, E. (2011). Quantitative contrast‐enhanced ultrasound imaging: A review of sources of variability. Interface Focus, 1, 520–539. 22866229 10.1098/rsfs.2011.0026 PMC3262271

[phy214580-bib-0051] Thijssen, D. H. , Rowley, N. , Padilla, J. , Simmons, G. H. , Laughlin, M. H. , Whyte, G. , Cable, N. T. , & Green, D. J. (2011). Relationship between upper and lower limb conduit artery vasodilator function in humans. Journal of Applied Physiology, 111, 244–250. 10.1152/japplphysiol.00290.2011 21512151 PMC3137536

[phy214580-bib-0052] Thomas, K. N. , Cotter, J. D. , Lucas, S. J. , Hill, B. G. , & van Rij, A. M. (2014). Reliability of contrast‐enhanced ultrasound for the assessment of muscle perfusion in health and peripheral arterial disease. Ultrasound in Medicine and Biology, 41, 26–34. 10.1016/j.ultrasmedbio.2014.06.012 25308937

[phy214580-bib-0053] Tonson, A. , Noble, K. E. , Meyer, R. A. , Rozman, M. R. , Foley, K. T. , & Slade, J. M. (2017). Age reduces microvascular function in the leg independent of physical activity. Medicine and Science in Sports and Exercise, 49, 1623–1630. 10.1249/MSS.0000000000001281 28709153 PMC5561656

[phy214580-bib-0054] Weber, M. A. , Krix, M. , & Delorme, S. (2007). Quantitative evaluation of muscle perfusion with CEUS and with MR. European Radiology, 17, 2663–2674. 10.1007/s00330-007-0641-y 17453217

[phy214580-bib-0055] Wei, K. , Jayaweera, A. R. , Firoozan, S. , Linka, A. , Skyba, D. M. , & Kaul, S. (1998). Quantification of myocardial blood flow with ultrasound‐induced destruction of microbubbles administered as a constant venous infusion. Circulation, 97, 473–483. 10.1161/01.CIR.97.5.473 9490243

[phy214580-bib-0056] Wen, W. , Luo, R. , Tang, X. , Tang, L. , Huang, H. X. , Wen, X. , Hu, S. , & Peng, B. (2015). Age‐related progression of arterial stiffness and its elevated positive association with blood pressure in healthy people. Atherosclerosis, 238, 147–152. 10.1016/j.atherosclerosis.2014.10.089 25485738

[phy214580-bib-0057] Wray, D. W. , Nishiyama, S. K. , Donato, A. J. , & Richardson, R. S. (2010). Human vascular aging: Limb‐specific lessons. Exercise and Sport Sciences Reviews, 38, 177–185. 20871234 10.1097/JES.0b013e3181f45413

